# Failed reinnervation in aging skeletal muscle

**DOI:** 10.1186/s13395-016-0101-y

**Published:** 2016-09-01

**Authors:** Sudhakar Aare, Sally Spendiff, Madhusudanarao Vuda, Daren Elkrief, Anna Perez, Qinghua Wu, Dominique Mayaki, Sabah N. A. Hussain, Stefan Hettwer, Russell T. Hepple

**Affiliations:** 1 Research Institute of the McGill University Health Centre, EM2.2232, RI MUHC, 1001 Decarie Blvd, Montreal, QC Canada H4A 3J1; 2 McGill Research Centre for Physical Activity and Health, McGill University, Montreal, QC Canada; 3 Department of Kinesiology and Physical Education, McGill University, Montreal, QC Canada; 4 Neurotune, Zurich, Switzerland

**Keywords:** Aging, Skeletal muscle, Denervation, Neurotrophins, MicroRNA, Muscle atrophy, Sarcopenia, Reinnervation

## Abstract

**Background:**

Skeletal muscle displays a marked accumulation of denervated myofibers at advanced age, which coincides with an acceleration of muscle atrophy.

**Methods:**

In this study, we evaluated the hypothesis that the accumulation of denervated myofibers in advanced age is due to failed reinnervation by examining muscle from young adult (YA) and very old (VO) rats and from a murine model of sporadic denervation secondary to neurotrypsin over-expression (Sarco mouse).

**Results:**

Both aging rat muscle and Sarco mouse muscle exhibited marked fiber-type grouping, consistent with repeating cycles of denervation and reinnervation, yet in VO muscle, rapsyn at the endplate increased and was associated with only a 10 % decline in acetylcholine receptor (AChR) intensity, whereas in Sarco mice, there was a decline in rapsyn and a 25 % decrease in AChR intensity. Transcripts of *muscle-specific kinase* (21-fold), acetylcholine receptor subunits *α* (68-fold), *ε* (threefold) and *γ* (47-fold), *neural cell adhesion molecule* (66-fold), and *runt-related transcription factor 1* (33-fold) were upregulated in VO muscle of the rat, consistent with the marked persistent denervation evidenced by a large proportion of very small fibers (>20 %). In the Sarco mice, there were much smaller increases in denervation transcripts (0–3.5-fold) and accumulation of very small fibers (2–6 %) compared to the VO rat, suggesting a reduced capacity for reinnervation in aging muscle. Despite the marked persistent denervation in the VO rat muscle, transcripts of neurotrophins involved in promoting axonal sprouting following denervation exhibited no increase, and several miRNAs predicted to suppress neurotrophins were elevated in VO rat.

**Conclusions:**

Our results support the hypothesis that the accumulation of denervated fibers with aging is due to failed reinnervation and that this may be affected by a limited neurotrophin response that mediates axonal sprouting following denervation.

**Electronic supplementary material:**

The online version of this article (doi:10.1186/s13395-016-0101-y) contains supplementary material, which is available to authorized users.

## Background

Skeletal muscle undergoes repeating cycles of denervation and reinnervation for much of adult life [1]. However, in very advanced age, when muscle atrophy becomes severe and has clinical impact in terms of impairing mobility [2], there is a marked accumulation of persistently denervated muscle fibers [3, 4]. Notably, we have shown in very old (VO) rat muscle that denervated myofibers are on average 35–50 % smaller than innervated fibers and innervated fibers of VO muscle are only 7 % smaller than in young adult [3]. The significance of this is that denervation is the primary cause of atrophy in the period of the lifespan when the clinical impact of aging muscle is most relevant. As an accumulation of persistently denervated fibers marks the acceleration of atrophy in both slow and fast twitch muscles with aging [5], understanding the mechanisms contributing to their accumulation is essential to provide effective targets for combating age-related muscle atrophy.

Although there have been a number of recent studies examining the mechanisms that may contribute to destabilization of the aging neuromuscular junction (NMJ) and subsequently lead to denervation of muscle endplates [6–8], there is scant information about the potential mechanisms contributing to impaired reinnervation of denervated fibers in very advanced age. Importantly, the progressive increase in fiber type grouping seen with increasing age [9] is clear evidence that following a sporadic denervation event, such fibers are usually successfully reinnervated for much of the lifespan. For this reason, a likely cause for the accumulation of persistently denervated fibers in very advanced age is a failure of reinnervation. In this context, previous studies have noted an impaired reinnervation response following acute nerve injury in aged animals that is due to impaired axonal regrowth [10, 11]. Despite this evidence, no prior studies have considered whether this impaired capacity for axonal regrowth (identified following acute nerve injury in aged animals) might also contribute to the accumulation of persistently denervated muscle fibers following denervation events that are part of the normal process of aging.

In addressing factors that might cause a compromised reinnervation response with aging, it is relevant to note that following surgical denervation in healthy young animals, there is a robust increase in neurotrophins, including *brain-derived neurotrophic factor* (*BDNF*) [12]. Furthermore, forced over-expression of the neurotrophins BDNF, *glial-derived neurotrophic factor* (*GDNF*) and *nerve growth factor* (*NGF*) in muscle facilitate axonal sprouting and reinnervation following surgical denervation [13]. Similarly, the exercise-induced enhancement of reinnervation following nerve crush injury is dependent upon muscle BDNF signaling [14]. As such, examination of neurotrophins involved in reinnervation and microRNAs (miRNAs) predicted to suppress neurotrophins could provide insights to the potential cause of failed reinnervation in very advanced age or at the least identify whether this pathway might represent a therapeutic target.

To help address these issues, we examined muscle morphology (fiber type and size distribution, fiber type grouping) and denervation-related transcripts (e.g., *muscle specific kinase* [*MuSK*], AChR *subunits*, *cytoplasmic phospholipase A2* [*cPLA2*], *neural cell adhesion molecule* [*NCAM*], and *runt-related transcription factor 1* [*RUNX1*]) in vastus lateralis (VL) muscle from young adult (YA; 8 months old) and very old (VO; 35 months old) male Fisher 344 × Brown Norway F1-hybrid (F344BN) rats and compared this to the same measures made in soleus and gastrocnemius muscles from male wild-type (WT) and neurotrypsin-over-expressing mice (Sarco mouse; a model of sporadic denervation). A key element of the study design is that the age of the Sarco mice (8 months of age) allows us to evaluate the capacity for reinnervation of young adult muscle subjected to sporadic denervation. We hypothesized that both VO rats and Sarco mice would exhibit marked fiber type grouping (indicating repeating cycles of denervation and reinnervation in both models) but compared to Sarco mice, VO rat muscle would exhibit a greater accumulation of small fibers indicative of persistent sporadic denervation and a commensurately greater expression of denervation-related transcripts. As both aging [9] and neurotrypsin over-expression [7] cause repeating cycles of denervation and reinnervation, a greater accumulation of sporadically denervated muscle fibers and up-regulation of denervation-related transcripts would implicate failed reinnervation as a major contributor to the accumulation of persistently denervated muscle fibers in advanced age. To provide insights to potential mechanisms of failed reinnervation in VO muscle, we also examined neurotrophin transcripts and miRNAs predicted to blunt neurotrophin levels.

## Results

### Fiber type proportions and fiber type grouping in aged rat and Sarco mice

**Fig. 1:**
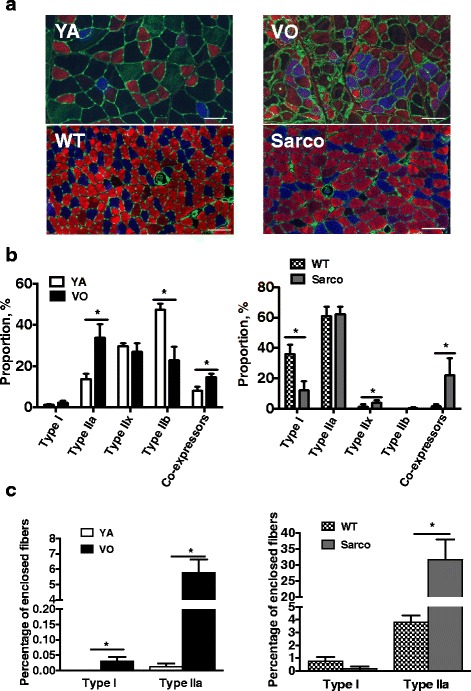
Muscle fiber type and type grouping. **a** Transverse muscle sections from rat vastus lateralis (VL) and mouse soleus muscle. Young adult (YA) vs very old (VO) rat VL muscle and wild-type (WT) vs Sarco mouse *soleus* were labeled with laminin to visualize the basal lamina and antibodies against the myosin heavy chains to visualize fiber types. *Scale bars* = 100 μm. Type I (*blue*), type IIa (*red*), type IIx (*green*), type IIb (*black* [no staining]) were labeled. **b** Fiber type proportions in YA vs VO rat and WT vs Sarco mouse. **c** Fiber type grouping in YA vs VO rat and WT vs Sarco mouse. In **b** and **c**, the data shown are means with standard error

Figure 1a shows representative images of the rat vastus lateralis and Sarco mouse soleus muscle cross-sections labeled using primary antibodies against myosin heavy chain (MHC) isoforms to identify fiber type and laminin to distinguish fiber borders. Proportions of pure fibers (types I, IIa, IIb, and IIx) were lower in the VO rat muscle compared to YA and lower in Sarco mice than wild-type mice. This decline in pure fibers was accounted for by an increase in abundance of fibers co-expressing two or more MHCs (MHC co-expressing fibers) in the VO rat and Sarco mice (Fig. 1b), a phenotype that we have shown previously to be largely secondary to denervation in the VO rat muscle [3]. To obtain an index of the occurrence of denervation-reinnervation events, we examined the abundance of fibers totally enclosed by fibers of the same type (fiber type grouping) in rat and mouse muscle. This analysis revealed that there was a large increase in fiber type grouping in both VO rat and Sarco mouse muscle (Fig. 1c).

### MuSK-rapsyn at the neuromuscular junction

**Fig. 2:**
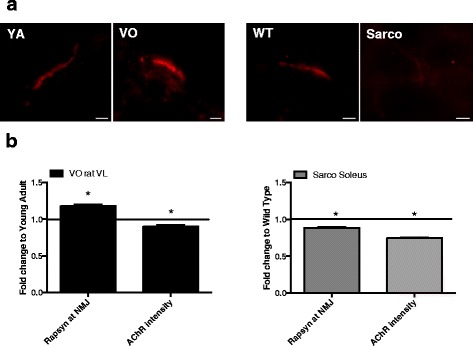
Protein analyses at the muscle endplate region. **a** Representative images of muscle endplates from YA vs VO (VL muscle) and WT vs Sarco (gastrocnemius muscle) labeled with primary antibodies against phospho-MuSK (*scale bars* = 10 μm). **b** Semi-quantitative analysis of protein intensities at the muscle endplate for rapsyn and AChRs in YA (*n* = 154–158 endplates) vs VO (*n* = 304–311 endplates) (VL muscle) and WT (*n* = 185 endplates) vs Sarco (*n* = 287–296 endplates) (gastrocnemius muscle). Values are normalized as a fold change of YA or WT, respectively. *P < 0.05 vs corresponding group within the same species

To evaluate changes in one of the primary signaling axes for regulating the integrity of the post-synaptic AChR plaque, we examined phospho-MuSK, rapsyn, and AChR intensity levels in the endplate region of the muscle fiber in both the aging and Sarco mice (Fig. 2). Interestingly, whereas immunofluorescent images show relatively preserved phospho-MuSK and semi-quantitative analysis in muscle cross-sections revealed an increase in rapsyn and only a small (10 %) decline in AChR intensity in VO rat muscle, phospho-MuSK appeared considerably reduced in Sarco Soleus muscle and this was accompanied by a decline in rapsyn and relatively large (25 %) decline in AChR intensity.

### Fiber size in aged rat and Sarco mice

**Fig. 3:**
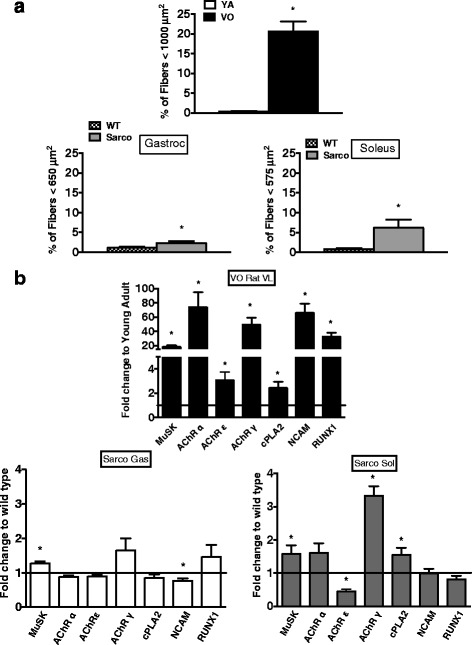
Morphological and transcriptional markers of denervation. **a** Abundance of very small fibers in YA vs VO rat vastus lateralis muscle, WT vs Sarco mouse gastrocnemius muscle, and WT vs Sarco mouse soleus muscle. **b** qPCR analysis of denervation markers: *MuSK*, *AChRa*, AChRe, *AChRg*, *cPLA2*, *NCAM*, and *RUNX1* in YA (*n* = 8) vs VO (*n* = 10) rat vastus lateralis muscle and WT (*n* = 8) vs Sarco mouse (*n* = 6) gastrocnemius and soleus muscle. *TBP* was used as endogenous control. Data are shown as means with standard error. *T* tests were performed. *P < 0.05 was considered as statistically significant

To gain insight into the frequency of failed reinnervation events in the VO rat, we examined the abundance of muscle fibers exhibiting a size of ≤1000 μm^2^, as these represent a population of muscle fibers shown by our group in VO rat gastrocnemius muscle to exhibit a high association with positive labeling for a marker of denervation (>90 % of these fibers are positive for the sodium channel isoform, Nav_1.5_) and to represent <1 % of fibers from young adult muscle [3]. We then compared this to a corresponding fiber size in Sarco mice (650 μm^2^ in gastrocnemius, 575 μm^2^ in soleus), based upon these representing a similar percentile of fiber size in the WT mice as in the YA rat. Interestingly, whereas VO rat exhibited >20 % of these very small fibers, Sarco mice exhibited approximately 2–6 % of these very small fibers (higher in soleus than gastrocnemius) (Fig. 3a). Taken in the context of the high frequency of denervation-reinnervation events evidenced by the large increase in fiber type grouping in both VO rat VL and Sarco mouse soleus muscle noted above (Fig. 1c), our analysis of very small fiber abundance reveals a marked impairment in reinnervation in VO rat muscle. On the other hand, a degree of compensatory hypertrophy was visually evident in photomicrographs of Sarco soleus muscle (Fig. 1a), consistent with a previous report [7].

### Transcriptional profile of denervation in aged rat and Sarco mice

The expression of denervation-related transcripts was dramatically increased in VL muscle of VO rat samples when compared to YA, and these increases were markedly greater than seen in Sarco mouse soleus or gastrocnemius muscles (Fig. 3b). Specifically, transcript levels of *MuSK*, a key signaling molecule involved in regulation of AChR clustering at the neuromuscular junction [15, 16], increased 21-fold in VO muscle (20–50 % change in Sarco mouse muscles). Similarly, transcript levels of the *AChR subunit α* (68-fold in VO vs YA) and *γ* (47-fold in VO vs YA) increased to a much greater degree in VO rat muscle than in Sarco mouse soleus muscle where *AChR* α had a borderline significant increase and *AChRγ* modestly increased (threefold) (no change in gastrocnemius muscle) vs wild-type mice. Interestingly, whereas AChR ε increased approximately threefold in VO rat VL muscle, it declined by nearly 60 % in Sarco soleus muscle. Transcript levels of *NCAM* (50-fold higher in VO vs YA), which encodes a protein that increases in aging muscle [17] and is involved in restoration of nerve contact to the muscle [18], and *RUNX1* (40-fold higher in VO vs YA), which encodes a protein that also increases in aging muscle [19] and counters muscle atrophy [20], were markedly elevated in VO rat muscle but not in Sarco mouse soleus or gastrocnemius muscle. Transcript levels of *cPLA*_*2*_, an enzyme catalyzing release of mitochondrial lipids as fatty acid hydroperoxides following denervation [21], increased in VO rat VL muscle and in Sarco mouse Soleus muscle (both approximately twofold relative to their respective controls). Collectively, these results identify a markedly greater denervation-related transcriptional profile in VO rat muscle than the Sarco mouse, despite both models being subjected to recurring cycles of sporadic denervation.

### Transcriptional profile of neurotrophins and related receptors in aged muscle

**Fig. 4:**
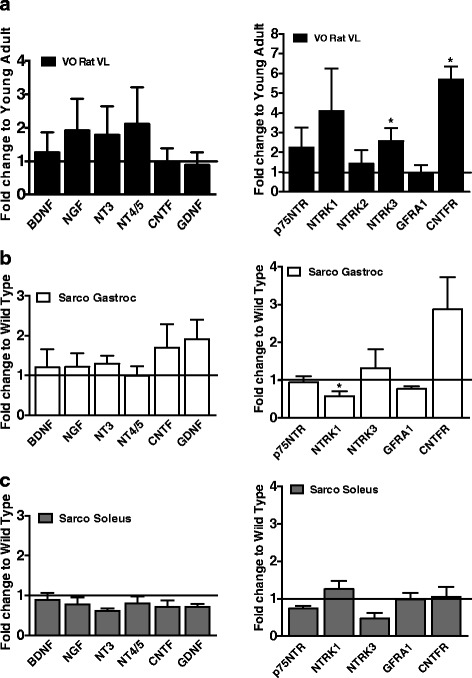
Neurotrophin and receptor transcript analysis. qPCR analysis of the neurotrophins: *BDNF*, *NGF*, *NT3*, *NT4/5*, *GDNF*, *CNTF* and neurotrophin receptors: *p75*, *NTRK1*, *NTRK2*, *NTRK3*, *GFRA1*, and *CNTFR* in YA (*n* = 8) vs VO (*n* = 10) rat VL (**a**), Sarco gastroc (**b**), and Sarco soleus (**c**) muscles. *TBP* was used as endogenous control. Data are shown as means with standard error. *T* tests were performed. *P < 0.05 was considered as statistically significant

The gene expression of neurotrophins (Fig. 4a left panel), including those implicated in promoting axonal growth following nerve injury [12, 13], were not significantly elevated in VO muscle, despite the marked accumulation of very small muscle fibers and large increases in expression of denervation-related transcripts. This was also the case in both muscles examined in Sarco mice (Fig. 4b, c left panels), although the denervation stress here is clearly less than with aging and there is likely less requirement for axonal sprouting due to the exclusively post-synaptic nature of the NMJ instability in this model. When considering transcripts for the neurotrophin receptors (Fig. 4b), only *neurotrophic tyrosine kinase receptor type 3* (*NTRK3*; encodes the *NT3 receptor*) and *ciliary neurotrophic factor receptor* (*CNTFR*; encodes the *CNTF receptor*) were increased in rat VO muscle. The other neurotrophin receptors, *p75NTR* (encoding the low affinity nerve growth factor receptor for several neurotrophins), *neurotrophic tyrosine kinase receptor type 1* (*NTRK1*; encoding the high-affinity receptor for *NGF*), *neurotrophic tyrosine kinase receptor type 2* (*NTRK2*; encoding the high-affinity receptor for *BDNF*), and *GDNF family receptor alpha-1* (*GFRA1*; encoding the *GDNF* receptor) exhibited no change in rat VO muscle compared to YA muscle (Fig. 3a, c). Similar to aging muscle, there were minimal changes in neurotrophin receptor transcripts in the Sarco mice, with only NTRK1 exhibiting a small decline in expression in Sarco mouse gastrocnemius muscle Fig. 4b, c left panels. Although this analysis does not reveal a decline in neurotrophin signaling in the VO muscle, taken in the context of the large increase in very small fibers and denervation-related transcripts, a reasonable conclusion is that the neurotrophin response is insufficient to facilitate successful reinnervation.

### Age-related changes in miRNAs predicted to suppress neurotrophins in aged muscle

**Fig. 5:**
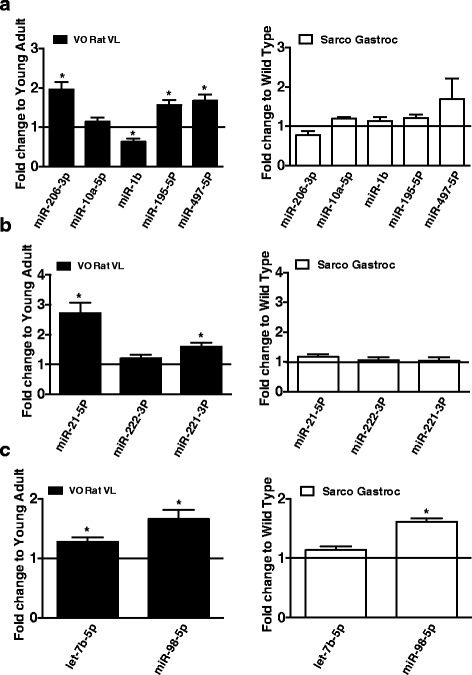
MicroRNA analysis. MicroRNAs predicted to influence neurotrophins: **a** *BDNF* (miR-206-3p, miR-10a-5p, miR-1b, miR-195-5p and miR-497-5p), **b** *NT3* (miR-21-5p, miR-222-3p and miR-221-3p), and **c** *NGF* (let-7b-5p and miR-98-5p) were quantified by qPCR analysis in YA (*n* = 8) vs VO (*n* = 10) rat vastus lateralis muscle and WT (*n* = 8) vs Sarco (*n* = 7) gastrocnemius muscle. miR-191a-5p was used as endogenous control. Data are shown as means with standard error. *T* tests were performed. *P < 0.05 was considered as statistically significant

Analysis of the *BDNF* 3′ untranslated region (UTR) using TargetScan (www.targetscan.org) revealed several target sites for *BDNF*: miR-206-3p, miR-10a-5p, miR-1b, miR-195-5p, and miR-497-5p that were conserved in humans. Both miR-206-3p and miR-1b have the same seed sequence (ACAUUCC). In VO rat muscle, miR-206-3p, miR-195-5p, and miR-497-5p were increased significantly. In contrast, miR-1b decreased and there was no change observed in miR-10a-5p (Fig. 5a). Similarly, TargetScan analysis identified the following miRNAs that are conserved in humans and are predicted to influence the *NT3* gene: miR-21-5p, miR-222-3p, and miR-221-3p. Both miR-222-3p and miR-221-3p share the same seed sequence (AUGUAGCA). In VO rat muscle, miR-21-5p and miR-221-3p were increased significantly (Fig. 5b). Finally, TargetScan identified the following miRNAs that are conserved in humans as likely to influence *NGF* transcript levels: let-7b-5p and miR-98-5p and these miRNAs share a common seed sequence (CUACCUCA). Both let-7b-5p and miR-98-5p were increased significantly in VO rat muscle (Fig. 5c). Thus, collectively, seven out of the ten miRNAs identified as likely candidates for suppressing neurotrophin expression were elevated in aging muscle. Of these, only miR-98-5p exhibited an increase in Sarco mouse gastrocnemius muscle (this analysis was not possible in soleus due to limited tissue).

## Discussion

There is a need for identification of viable therapeutic targets to treat aging muscle in advanced age where the clinical impact of muscle atrophy becomes meaningful. A key step in this endeavor is a clear understanding of the processes that lead to muscle atrophy in advanced age. To this end, the purpose of this study was to evaluate the hypothesis that the accumulation of persistently denervated muscle fibers in advanced age is due to failed reinnervation by comparing changes with aging to changes seen in a mouse model of sporadic denervation, the Sarco mouse.

Our results show that VO rat VL muscle is characterized by marked accumulation of very small fibers and MHC co-expression, two hallmark features of sporadic denervation in aging muscle [3]. Although there are many reasons fiber atrophy could occur, its sporadic nature (atrophied fibers interspersed amongst relatively normal sized fibers) in VO muscle is also seen in neuromuscular diseases that exhibit denervation, such as amyotrophic lateral sclerosis and spinal muscular atrophy [22]. Further, to this point, although MHC co-expression can also occur in the context of sedentary lifestyle with aging and is reduced in response to exercise training [23], a distinguishing feature in VO rat muscle is that MHC co-expression most often co-localizes with other features of denervation, including small and angular fiber size and Nav_1.5_ expression [3]. Furthermore, VO rat muscle exhibited marked fiber type grouping (indicative of many repeating cycles of denervation and reinnervation [9]) and large induction of transcripts known to respond to denervation. In contrast, despite significant fiber type grouping in Sarco mouse muscle and a high abundance of MHC co-expressing fibers (suggesting that, similar to normal aging, Sarco mice also undergo many repeating cycles of denervation and reinnervation), the abundance of small fibers and induction of denervation-related transcripts compared to WT mice was much smaller than seen with aging. This analysis suggests a robust reinnervation response in young muscle (evidenced by the Sarco mouse) and in turn a poor reinnervation response of aging muscle. In identifying a possible mechanism for failed reinnervation, our analysis suggests that an insufficient neurotrophin response in aging muscle may contribute to the persistence of denervated muscle fibers and that this may be attributable to increases in miRNAs that suppress neurotrophin expression in aging muscle. The translational significance of these observations is that they suggest that up-regulation of neurotrophin signaling could be an effective therapeutic approach for attenuating muscle atrophy by facilitating reinnervation in advanced age where muscle impact is clinically meaningful.

### The evolving causes of atrophy in aging muscle

Whereas at ages ≤75 years old muscle fibers atrophy relatively uniformly [24], in more advanced ages where the severity of muscle atrophy is more likely to have clinical impact [2], the muscle morphology is punctuated by a marked accumulation of small angular fibers that are interspersed amongst relatively normal-looking muscle fibers [4]. Identification of processes that can lead to sporadic fiber atrophy in aging muscle is thus key to understanding the causes of muscle atrophy in advanced age. Although segmental accumulation of mitochondrial defects has been considered a cause of muscle fiber atrophy with aging [25], the fraction of fibers harboring mitochondrial defects that exhibit atrophy is very small (5 %) [5, 26] and thus unlikely to represent a key target. On the other hand, we have shown that there is a marked accumulation of small angular fibers that coincides with the acceleration of muscle atrophy in both slow and fast twitch muscles in advanced aging [5] and that >90 % of these fibers label positively for a sodium channel isoform, Nav_1.5_ [3], that is upregulated in response to denervation [27]. It is for this reason that we have focused the current study on identifying mechanisms that could account for the accumulation of persistently denervated muscle fibers in aging muscle.

### Sarco mouse as a model of sporadic denervation

In our studies, we used a genetic model of sporadic denervation, the Sarco mouse [28]. This model was engineered to over-express the endogenous protease, neurotrypsin, in neurons which in turn causes increased agrin cleavage at the neuromuscular junction and reduces activation of MuSK [7]. The result is less recruitment of rapsyn to the subsynaptic microdomain, a resulting loss of cytoskeletal tethering of the post-junctional AChRs, and thus, an increased susceptibility to sporadic denervation. Although we present data from both gastrocnemius and soleus muscles, our analysis shows that for the parameters examined, the changes are generally greater in the soleus muscle. For example, there was a greater increase in fraction of very small muscle fibers and larger changes in denervation-related transcripts in the relatively slow twitch postural soleus muscle compared to the largely fast twitch gastrocnemius muscle in the Sarco mice. Thus, whilst fiber type and muscle recruitment are clearly different between rat VO and mouse Soleus muscles, the Sarco mouse soleus is more affected and might on this basis provide a better indicator of the degree to which reinnervation is compromised with aging.

Consistent with prior studies using this model [7, 28], the Sarco mice exhibited smaller muscle mass and a pronounced fiber type shift punctuated by a large accumulation of MHC co-expressing fibers. Furthermore, phospho-MuSK levels at the NMJ were seen to decrease (Fig. 2a), as would be expected in response to reduced agrin activation of MuSK [7], and this was accompanied by a reduced rapsyn and AChR intensity in the post-synaptic endplate region. In addition, we observed marked fiber type grouping (only examined in soleus muscle) and moderate increases in some denervation-related transcripts (MuSK in both muscles, and *AChRγ* and *cPLA2* in soleus muscle). We also noted a nearly 60 % decrease in *AChR ε* subunit in Sarco soleus muscle, which contrasts with the increase in *AChR ε* that was seen in VO rat muscle. In this respect, previous studies have shown that following denervation *AChR ε* expression undergoes a multiphasic response: (i) an initial decrease in the first 10 day post-denervation [29]; (ii) a sharp increase observed approximately 4 weeks following denervation; (iii) a decline over the next 4 weeks; and (iv) a gradual secondary increase when denervation persists beyond 2 months [30, 31]. Thus, the differences between the VO rat and Sarco mouse likely reflect differences in the duration of denervation, with the higher *AChR ε* in VO rat reflecting more prolonged denervation and the reduced *AChR ε* in Sarco mouse reflecting the acute denervation response.

A caveat to comparing the Sarco mouse to aging muscle concerns the frequency of sporadic denervation events. The large increase in frequency of enclosed fibers (Fig. 1c) shows both are exposed to many recurring cycles of denervation and reinnervation. Accepting that it is not possible to obtain a quantitative comparison of the exact frequency of denervation (e.g., on a daily or weekly basis) events in the two models, the value of the Sarco mouse herein is that it provides an indicator of the normal capacity for reinnervation of young adult muscle in the context of significant neuromuscular junction instability, and as such, suggests a compromised reinnervation capacity of VO muscle. On the other hand, we cannot rule out that the aging muscle might undergo a higher frequency of denervation events, which is itself the problem (rather than failed reinnervation per se). As noted above, however, the *AChR ε* response in the VO rat is more consistent with prolonged persistent denervation rather than a greater burden of denervation per se.

### Causes of denervation and failed reinnervation in aging muscle

Skeletal muscle undergoes repeating cycles of denervation and reinnervation throughout much of the adult life, resulting in remodeling of the aging motor unit that in turn causes significant grouping of fibers of the same type [9]. The causes of individual denervation events with aging may involve gradual changes in the signaling pathways necessary for NMJ maintenance and/or a sudden change such as muscle injury or motor neuron loss [32]. In our aging rat model, we have previously shown in the same animals studied here that there is a 27 % loss of motor neuron cell bodies in the spinal cord [3], indicating a significant pre-synaptic element causing denervation in VO rat muscle. This is in contrast to the Sarco mouse where the defect is downstream of the motor neuron and where motor neuron number in the spinal cord is maintained [7]. Thus, although both models exhibit denervation, the causes may be quite different. This notion is supported by the fact that in our study, phospho-MuSK was maintained in aging muscle (decreased in Sarco mouse), rapsyn was increased (reduced in Sarco mouse), and this resulted in only a small loss of AChR intensity in the post-synaptic endplate region of aging muscle (much larger reduction in AChR intensity in Sarco mouse).

Consistent with other studies that have examined transcriptional responses in aging muscle [19, 33], in VO rat muscle, we observed increases in several transcripts that change with denervation. The large magnitude of these changes underscores the very significant denervation stress present in the VO muscle, and this contrasts markedly with the much smaller changes seen in Sarco mouse muscles. Notably, previous studies have shown that the neurotrophins *BDNF*, *NGF*, and *GDNF* are important to promote motor axon sprouting and recovery of innervation following surgical denervation [12, 13, 34]. Furthermore, interventions that increase signaling through BDNF and/or its receptor TrkB markedly improve recovery of diaphragm function following cervical spinal cord injury [35–37]. Yet, we observed largely non-significant increases in the neurotrophin and neurotrophin receptor transcripts in VO rat muscle which could indicate a failed response to the marked presence of sporadically denervated fibers in aging muscle. This notion is consistent with previous studies showing impaired BNDF signaling in aging limb muscle [38] and diaphragm [39]. In contrast to aging muscle, the fact that motor neurons are spared in neurotrypsin-over-expressing mice [7] would obligate less extensive axonal sprouting to facilitate reinnervation in this context. Thus, the negligible changes in neurotrophins and their receptors in Sarco mouse muscles are not unexpected. It follows that a significant limitation to our interpretation of the neurotrophin response in VO muscle is that we do not have a model to indicate the “normal” adult neurotrophin response to sporadic motor neuron loss. Such information could be obtained from either experimental models involving a less severe form of denervation involving partial nerve crush or a genetic model involving motor neuron loss. Regardless of this limitation, on the basis of the aforementioned evidence showing the importance of neurotrophins in promoting axonal sprouting and reinnervation following experimental denervation, therapeutically targeting the neurotrophins in advanced age is worthy of evaluation.

### Potential involvement of miRNA-induced suppression of neurotrophins

There has been remarkable progress in recent years in identification of miRNAs, opening up new avenues of research into mechanisms of disease [40, 41], as well as mechanisms that can contribute to muscle impairment in various disorders [42, 43]. Although it is well established that miRNAs can suppress gene expression by pairing with target mRNAs to direct their degradation or influence the mRNA translation capacity [44], very few studies have examined miRNAs in the context of aging muscle [45, 46], and none specifically in the context of the causes of persistent denervation. Thus, in seeking mechanisms that might account for a blunted neurotrophin response to denervation in aging muscle, we first used TargetScan to identify potential miRNAs that could suppress neurotrophins. This analysis identified 10 miRNAs that are conserved in humans and which target three neurotrophins involved in promoting axonal sprouting following nerve injury. For *BDNF*, our analysis identified increases in miR-206-3p, miR-195-5p, and miR-497-5p in VO rat muscle. Similarly, for *NT3*, our analysis identified significant increases in miR-21-5p and miR-221-3P in VO rat muscle. Finally, for *NGF*, our analysis revealed an increase in both let-7b-5p and miR-98-5p in VO rat muscle. Of these, miR-206 has previously been shown to increase in aging mouse muscle [46], consistent with our analysis in VO rat muscle. Similarly, miR-195 (predicted to target *BDNF*), and let-7b and miR-98 (both predicted to target *NGF*) have been shown previously in human muscle to increase with aging [45], consistent with our analysis in VO rat muscle. To our knowledge, none of the other miRNAs that we identified as potentially suppressing neurotrophins have been examined previously in the context of aging muscle.

In contrast to the aforementioned studies in aging muscle, most of the miRNAs studied herein have been examined in the context of experimental denervation, including miR-206 (increases after reinnervation), miR-10a-5p (increases four- to seven-fold with denervation), miR-1 (increases up to 10-fold following denervation and remains elevated after reinnervation), miR-195 (increases up to 10-fold with denervation), miR-21 (increases with denervation), miR-221 (no consistent change), miR-222 (no consistent change), and miR-98 (increases up to 10-fold with denervation) [43, 47, 48]. Note that we also saw an increase in miR-98-5p in Sarco mouse muscle, which likely reflects a normal response to denervation. Thus, the normal response to experimental denervation, and in the current study, a model of NMJ instability (Sarco mouse), is often an increase in miRNAs that are predicted to target neurotrophin genes. The miRNAs that exhibited a different response in VO rat muscle from what is seen with experimental denervation are miR-1, which in experimental denervation increases [48] but in VO rat muscle was observed to decline, and miR-221, which does not change with denervation [47] but was observed to increase in VO rat muscle. Since miRNAs frequently target more than a single mRNA species, a simple interpretation of the biological significance of the changes observed is challenging. Thus, whether miRNA sponges [41] or other approaches to silencing miRNA signaling would be effective in promoting reinnervation in aging muscle by amplifying the neurotrophin response, will need experimental validation.

## Conclusions

The purpose of our study was to evaluate whether the marked accumulation of persistent denervated fibers in muscle at advanced age is due to failed reinnervation. Consistent with our hypothesis, VO rat muscle exhibited a much greater accumulation of very small muscle fibers and denervation-related transcripts than Sarco mouse muscle, even though both models evidenced marked fiber type grouping indicative of recurring cycles of denervation and reinnervation. In spite of this significant denervation stimulus in VO rat muscle, the neurotrophin pathways known to drive axonal sprouting following acute nerve injury exhibited a minimal induction (with most changes not reaching statistical significance). We conclude that failed reinnervation is an important mechanism driving the acceleration of muscle atrophy in advanced age and that therapeutically targeting the neurotrophin response to stimulate axonal sprouting would be worthy of exploration.

## Methods

### Experimental procedures

#### Animals and tissue collection

We studied 8-month-old (young adult; YA; *n* = 8) and 35–36-month-old (very old; VO; *n* = 10) male Fischer 344 X Brown Norway F1-hybrid (F344BN) rats obtained from the National Institute on Aging (USA) colony. Upon arrival at the University of Calgary Biological Sciences vivarium, the animals were housed individually in cages fitted with filter bonnets and maintained in a 12:12-h light/dark cycle at 23 °C for at least 48 h before experiments. On the day of experiments, the animals were anesthetized with 55–65 mg kg^−1^ of sodium pentobarbital and the left vastus lateralis muscle was removed, trimmed of adipose and connective tissue, and weighed. A slice through the entire midbelly of the vastus lateralis muscle was mounted transversely on pieces of cork using optimal cutting temperature compound and frozen in isopentane pre-cooled in liquid nitrogen before being stored at −80 °C. Remaining muscle portions were frozen in liquid nitrogen for RNA and microRNA measurements and stored at −80 °C. All procedures with F344BN rats were conducted with approval from the Animal Care Committee at the University of Calgary (ID B109R-11).

Neurotrypsin over-expressing C57BL/6 mice were provided by Neurotune, Switzerland, and bred at the Research Institute of the McGill University Health Centre vivarium. All of the mice were maintained in a heterozygous state and were backcrossed with wild-type C57BL/6 mice. In all experiments, 8-month-old mice were used to study the impact of sporadic denervation and the fidelity of reinnervation in young adult muscle. On the day of the experiment, the animals were sacrificed with CO_2_ asphyxiation followed by cervical dislocation. The right soleus and gastrocnemius muscles were dissected free of adipose and connective tissue and weighed, and a portion of each was mounted in tragacanth gum and frozen in isopentane pre-cooled in liquid nitrogen. The remaining portion of each muscle was frozen in liquid nitrogen for mRNA and miRNA (gastrocnemius muscle only in Sarco mouse) analyses. All experimental procedures with mice were conducted with approval from the Animal Care Committee at McGill University Health Centre.

#### Muscle sectioning

Ten-micrometer-thick cross-sections were cut using a Leica CM-3050-S cryostat (−20 °C) and mounted onto glass slides. The sections were left to air-dry for 2 h before being stored at −80 °C until use. Prior to labeling protocols (see below), the sections were defrosted in slide boxes for 30 min and allowed to air dry.

#### Fiber type, size, and proportion assessments

Muscle cross-sections were immunolabeled for myosin heavy chains (MHCs) I, IIa, IIx, and IIb by a previously described method [24]. The sections were rehydrated with PBS (pH 7.2). These sections were blocked using goat serum (10 % in PBS) and incubated for 1 h at room temperature with the following primary antibody solution (unless otherwise stated, from the Developmental Studies Hybridoma Bank, Iowa, USA): mouse IgG2b monoclonal anti-MHC type I (BA-F8, 1:25), mouse IgG1 monoclonal anti-MHC type IIa (SC-71, 1:200), mouse IgM monoclonal anti-MHC type II× (6H1, 1:25), mouse IgM monoclonal anti-MHC type IIb (BF-F3, 1:200), and a rabbit IgG polyclonal anti-laminin (ThermoFisher Scientific). The muscle sections were then washed three times in PBS before being incubated for 1 h at room temperature with the following secondary antibodies (all obtained from ThermoFisher Scientific): Alexa Fluor 350 IgG2b (y2b) goat anti-mouse (A-21140, 1:500), Alexa Fluor 594 IgG1 (y1) goat anti-mouse (A-21125, 1:100), Alexa Fluor 488 IgM goat anti-mouse (A-21042, 1:500), and Alexa Fluor 488 IgG goat anti-rabbit (A-11008, 1:500) diluted in blocking solution. Due to the presence of four MHC isoforms in rodent muscle, labeling was performed on two serial sections (section 1: three MHC antibodies + laminin, section 2: one MHC antibody + laminin). Muscle cross-sections were then washed three times in PBS, and coverslips were applied to slides using Prolong Gold (ThermoFisher Scientific; P36930) as mounting medium. The slides were imaged with a Zeiss fluorescence microscope (Axio Imager, Zeiss, Oberkochen, Germany). Frames for analysis were systematically sampled across each muscle section. The frequency of very small fibers was assessed as an index of the degree of persistent denervation in both aging rat and Sarco mice, based upon previous data from our group showing that fibers of this size in VO rat gastrocnemius muscle exhibit >90 % positive labeling for a marker of denervation, the sodium channel isoform Nav_1.5_ [3]. Specifically, we used the fiber size corresponding to the first percentile in YA (for rat) or WT (for mice) animals to define very small fiber size. The first percentile was determined by calculating the size distribution of all fibers for all animals in either YA rats or WT mice. In VO rat VL muscle, this corresponded to fibers with a size of ≤1000 μm^2^, whereas in Sarco mouse, this corresponded to fibers with a size of 650 μm^2^ in gastrocnemius muscle and ≤575 μm^2^ in soleus muscle.

#### Neuromuscular junction labeling

We first labeled sections to identify levels of activated MuSK based upon phospho-MuSK staining intensity. Tissue cross-sections of rat VL and mouse gastrocnemius muscle were rehydrated with PBS and then fixed in 2 % paraformaldehyde containing Phosphatase Inhibitor Cocktail tablet for 10 min at room temperature and then washed (4 × 5 min PBS, 1× PBS) before being incubated in permeabilization solution (0.2 % Tween 20 in PBS) at room temperature for 10 min. The sections were washed again and incubated in blocking solution (10 % NGS) for 60 min. Incubation with anti-MuSK (phospho Y755) antibody (1:250 in blocking buffer; Rabbit polyclonal, Abcam; ab192583) was performed overnight at 4 °C in a humidified chamber. The slides were washed and then incubated with AF594 IgG goat anti-rabbit (ThermoFisher Scientific, A11037; 1:100 in blocking buffer) antibody for 1 h at room temperature. The sections were washed a final time and then mounted with ProLong Gold Antifade Mountant (ThermoFischer Scientific). Images were captured on an Axio Imager M2 (Zeiss) microscope using ×40 objective.

We next examined the downstream impact of alterations in MuSK activation by examining muscle endplate levels of the AChR tethering protein, rapsyn, and AChR intensity levels. Tissue cross-sections of rat VL and mouse gastrocnemius muscle were fixed in acetone for 15 min at 4 °C then washed (2 × 5 min PBST, 1× PBS) before being incubated in permeabilization solution (0.1 % Triton in PBS) at room temperature for 15 min. The sections were washed again and incubated in blocking solution (1 % NGS 5 % BSA) for 30 min. Incubation with α-bungarotoxin 488 conjugate (ThermoFisher Scientific; B13422) and rapsyn (mouse monoclonal, Abcam; Ab11423) was performed at room temperature for 2 h in a humidified chamber, where all dilutions were made up in blocking solution. The slides were washed, and AF350 IgG1 goat anti-mouse (ThermoFisher Scientific 1:100) was applied for 1 h at room temperature. The sections were washed a final time and then mounted with ProLong Gold Antifade Mountant (ThermoFischer Scientific). Images were captured on an Axio Imager M2 (Zeiss) and then de-convoluted on Autoquant X3 using the “blind deconvolution” setting and image parameters provided by the Axio imager M2 metadata. Analysis of NMJ labeling was performed using ImageJ (National Institutes of Health, Bethesda), with NMJs being traced semi-automatically and the investigator blinded to the group status of the animal.

### Gene expression

Total RNA was extracted from YA and VO rat vastus lateralis muscle samples using the RNeasy Lipid Tissue Mini Kit (Qiagen; 74804). Quantification and purity of total RNA was assessed with a nanodrop by examining the A260/A280 absorption ratio. RNA (1 μg) was reverse transcribed to cDNA using qScript*™* cDNA Synthesis Kit (Quanta biosciences; 95047-025), according to the manufacturer’s instructions. Real-time PCR was performed using a StepOnePlus™ Real-Time PCR system (ThermoFisher Scientific) to quantify the different denervation-related genes (*Musk*, *AChRα*, *AChRγ*, *cPLA2*, *NCAM*, and *RUNX1*) (Additional file 1: Table S1) and neurotrophin-related genes (*BDNF*, *NGF*, *GDNF*, *CNTF*, *p75NTR*, *NTRK1*, *NTRK2*, *NTRK3*, *GFRA1*, *CNTFR*) (Additional file 1: Table S2). *TATA box binding protein* (*TBP*) (endogenous control) was obtained from RealTimePrimers.com. Primers were designed with a freely available software, Primer 3 plus. Power SYBR*®* Green PCR Master Mix (ThermoFisher Scientific; 4367659) was used to quantify the mRNA. The thermal profile was 95 °C for 10 min and 40 cycles of 95 °C for 15 s and 55–60 °C for 60 s. All real-time PCR experiments were performed in triplicate, and melt curve analysis for each PCR experiment was performed to assess primer dimer formation or contamination. The comparative threshold cycle (CT) method was used to calculate fold changes in expression. Relative foldchanges in gene expression were presented as 2−ΔΔCT and normalized to YA (F344BN rats) or WT (Sarco mice) values.

### MicroRNA analysis

Freely available target scan software (www.targetscan.org) was used for predicting microRNAs targeting *BDNF*, *NT3*, and *NGF* genes. Subsequently, we used the software miRBase (www.mirbase.org) to obtain the sequence of the different miRNAs predicted to target these neurotrophin genes. Total RNA was extracted from both YA and VO rat VL muscle using *mir*Vana™ miRNA Isolation Kit, with phenol (Thermo Fisher Scientific Inc; AM1560), according to the manufacturers’ protocols. MiRNAs were detected using an NCodeTM miRNA qRT-PCR Kit (ThermoFisher Scientific; 45-6612) and real-time PCR (StepOnePlus™ Real-Time PCR system, Thermal Cycling Block; ThermoFisher Scientific) with specific primers (Additional file 1: Table S3), Platinum® SYBR® Green qPCR SuperMix-UDG (ThermoFisher Scientific; 11733-038). All experiments were performed in triplicate. Relative miRNA expression was determined using the CT method, where CT values of individual miRNA data were normalized to CT values of miR-191a-5p. (Fig. 5)

### Statistical analysis

GRAPHPAD PRISM (GraphPad Software, Inc. La Jolla, CA, USA) software was used for statistical analysis. All statistical analyses involved an unpaired two sided *T* test when comparing two groups. Fiber type distribution was analyzed by two-way ANOVA (fiber type x group), with a Sidak’s post hoc test. The outliers were detected by using GraphPad prism Grubbs’ test. *P* < 0.05 was considered as statistically significant.

## Additional file

Additional file 1: Table S1. DNA sequences and primers for qPCR analyses of neurotrophins and neurotrophin receptors in rat skeletal muscle. Table S2. DNA sequences of primers used for qPCR analyses of neurotrophins and neurotrophin receptors in mouse muscle. Table S3. microRNA sequences of primers used to perform QPCR experiments to detect miR levels in rat vastus lateralis and mouse gastrocnemius muscle. (PPTX 1239 kb)

## Abbreviations

AChR: Acetylcholine Receptor
BDNF: Brain-derived Neurotrophic Factor
CNTF: Ciliary Neurotrophic Factor
CNTFR: CNTF Receptor
cPLA2: Cytoplasmic Phospholipase A2
GDNF: Glial Derived Neurotrophic Factor
GFRA1: GDNF Family Receptor Alpha-1
MHC: Myosin Heavy Chain
miRNA: MicroRNA
MuSK: Muscle Specific Kinase
Nav_1.5_: Sodium Channel Isoform 1.5
NCAM: Neural Cell Adhesion Molecule
NGF: Nerve Growth Factor
NMJ: Neuromuscular Junction
NTRK1: Neurotrophic Tyrosine Kinase Receptor 1
NTRK2: Neurotrophic Tyrosine Kinase Receptor 2
NTRK3: Neurotrophic Tyrosine Kinase Receptor Type 3
p75NTR: p75 Neurotrophin Receptor
phospho-MuSK: Phosphorylated MuSK
RUNX1: Runt-related Transcription Factor
Sarco: Neurotrypsin Over-expressing Mouse
UTR: Untranslated Region
VL: Vastus Lateralis
VO: Very Old
WT: Wild Type
YA: Young Adult
