# Supplementary material for: Failed reinnervation in aging skeletal muscle
Source: Skelet Muscle. 2016 Sep 1;6(1):29. doi: 10.1186/s13395-016-0101-y (PMC5007704; doi:10.1186/s13395-016-0101-y)
Supplement: Additional file 1: Table S1. — DNA sequences and primers for qPCR analyses of neurotrophins and neurotrophin receptors in rat skeletal muscle. Table S2. DNA sequences of primers used for qPCR analyses of neurotrophins and neurotrophin receptors in mouse muscle. Table S3. microRNA sequences of primers used to perform QPCR experiments to detect miR levels in rat vastus lateralis and mouse gastrocnemius muscle. (PPTX 1239 kb) [file 13395_2016_101_MOESM1_ESM.pptx]

## Slide 1
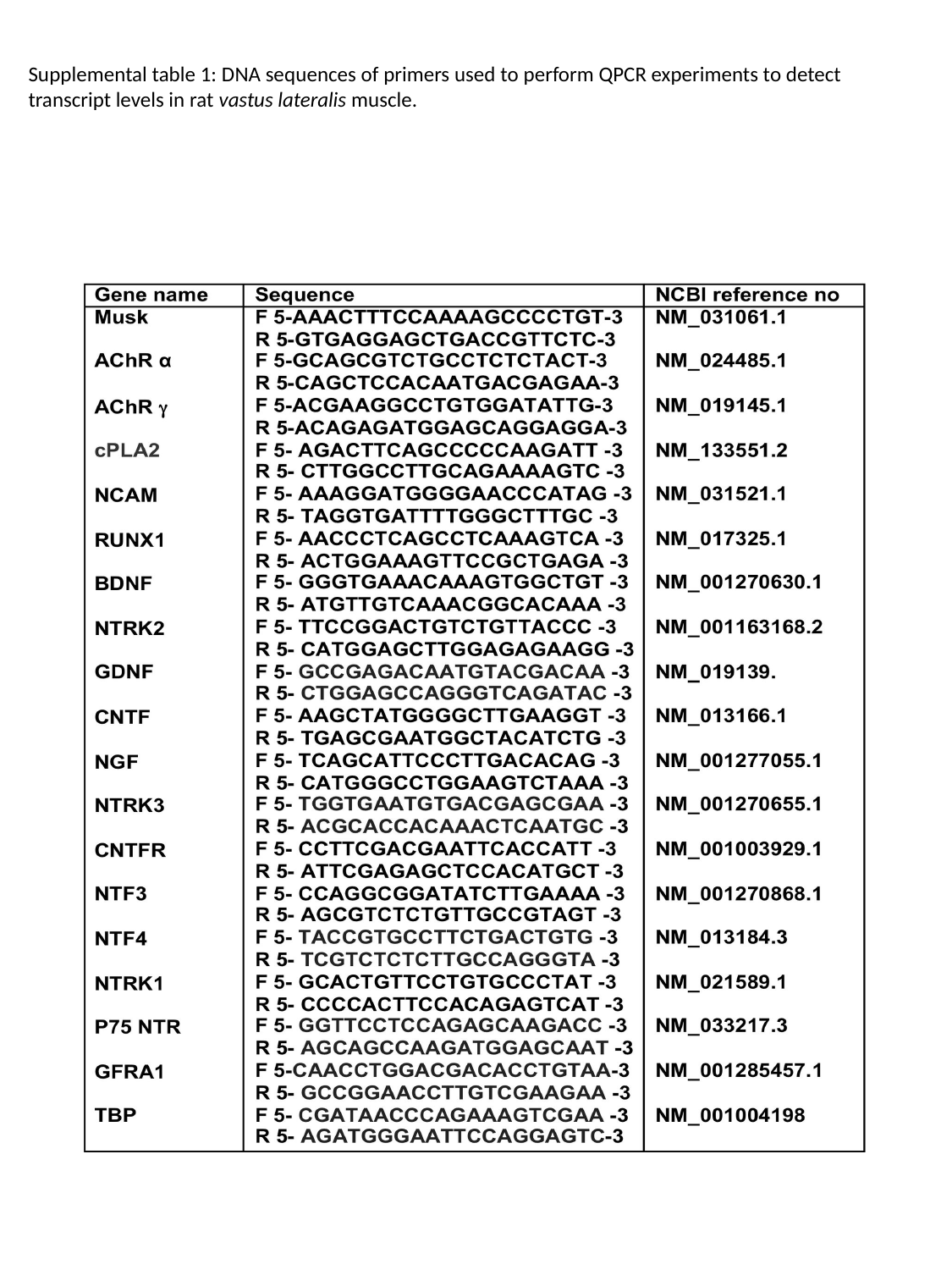

Supplemental table 1: DNA sequences of primers used to perform QPCR experiments to detect transcript levels in rat vastus lateralis muscle.

## Slide 2
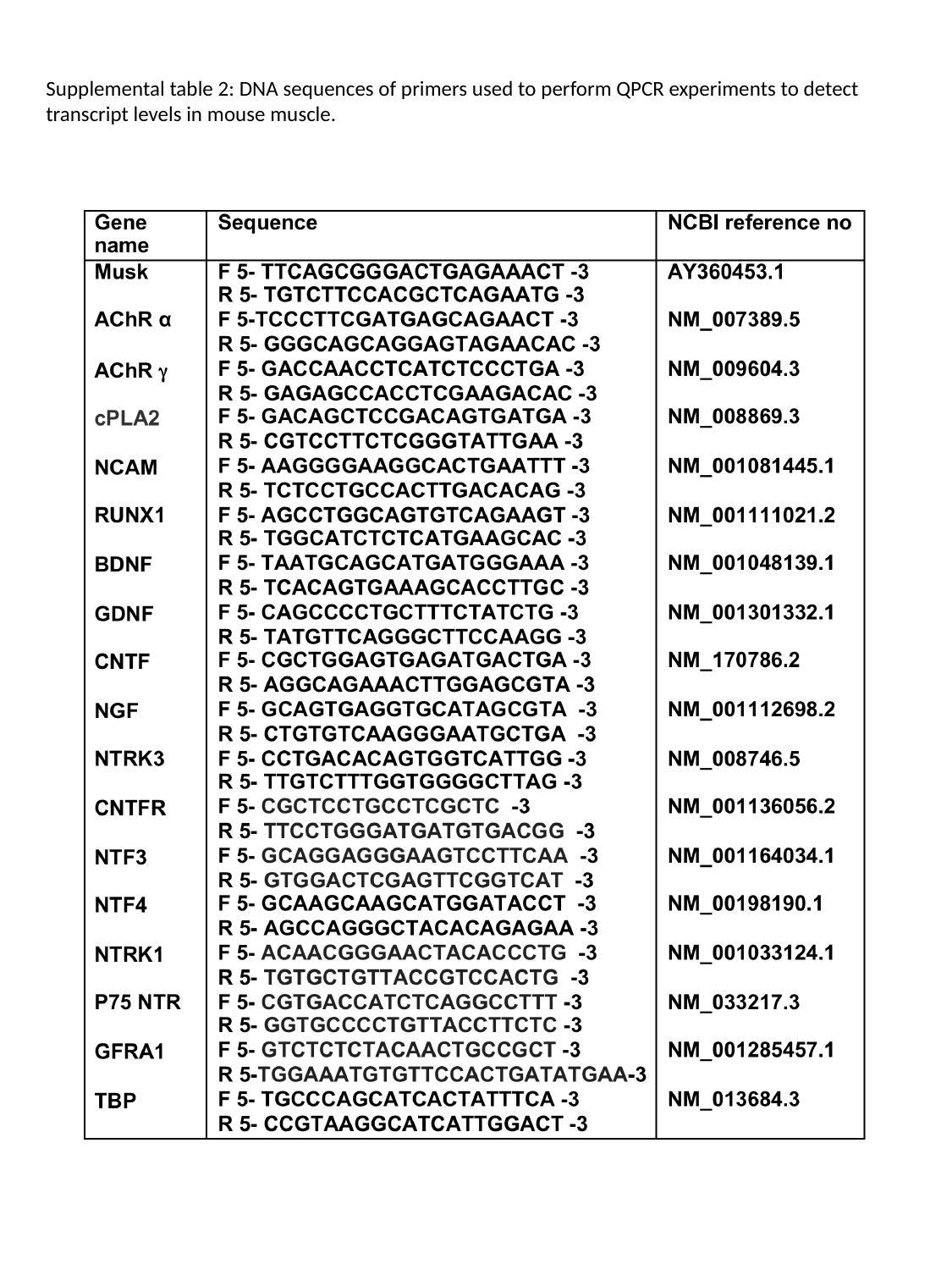

Supplemental table 2: DNA sequences of primers used to perform QPCR experiments to detect transcript levels in mouse muscle.

## Slide 3
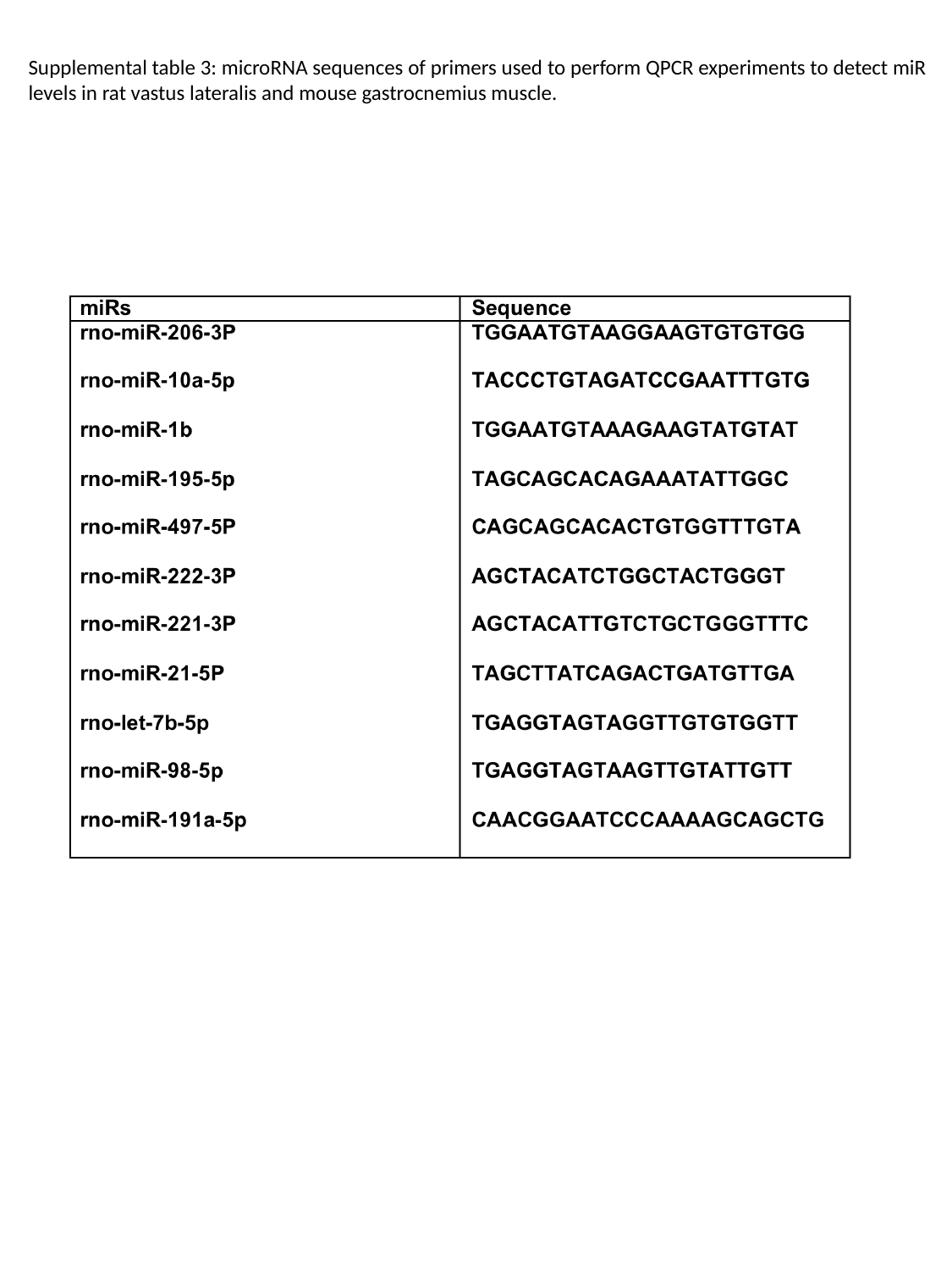

Supplemental table 3: microRNA sequences of primers used to perform QPCR experiments to detect miR levels in rat vastus lateralis and mouse gastrocnemius muscle.
